# Editorial: Positive or negative? The effect of emerging technologies and products on mental health

**DOI:** 10.3389/fpsyt.2023.1282385

**Published:** 2023-09-11

**Authors:** Chao Guo, Stuart Gilmour, Peige Song, Aiping Fang

**Affiliations:** ^1^Institute of Population Research, Peking University, Beijing, China; ^2^Graduate School of Public Health, St. Luke's International University, Tokyo, Japan; ^3^School of Public Health, School of Medicine, Zhejiang University, Hangzhou, Zhejiang, China; ^4^Guangdong Provincial Key Laboratory of Food, Nutrition and Health, Department of Nutrition, School of Public Health, Sun Yat-sen University, Guangzhou, China

**Keywords:** digital, technology - ICT, internet of things (IoT), mental health, effect

In recent years, rapid advances in digital health technology, computing platforms, and the internet of things (IoT) have revolutionized the way we interact, communicate, and access information. These innovations have the potential to empower individuals and enhance societal wellbeing, as well as revolutionize healthcare delivery. However, they also bring new challenges, especially concerning their impact on mental health.

Numerous studies have explored the relationship between emerging technologies and mental health, leading to a dynamic and ongoing debate. Some research suggests that excessive use of social media may be associated with adverse mental health outcomes, including depression, anxiety, cyberbullying, and social isolation (1–3). There is particular concern about the potential negative effects of digital technology on mental wellbeing among adolescents and young adults (4). Conversely, other scholars argue that digital technology products can also be leveraged to address mental health issues in innovative and effective ways (5), and that they have great promise in providing timely, personalized, and accessible interventions for various mental health conditions (6).

In light of the pervasive presence of emerging technologies and their intricate interplay with mental health, further research is essential to harness their potential, while addressing potential mental health challenges emerging alongside the widespread integration of digital and electronic devices in contemporary society. Delving into the intricate relationship between emerging technologies and mental wellbeing holds significance beyond merely gauging potential advantages and drawbacks; it also serves as a foundation for crafting precise interventions and fostering psychological health.

To contribute to this vital area of research, this Research Topic presents a collection of nine articles that delve into the positive and negative effects of emerging technologies and products on mental health. These articles shed light on various aspects of the relationship between digital technology use and mental wellbeing.

Firstly, this Research Topic focuses on the potential mental health impacts of the most ubiquitous digital technology, the Internet. Nie et al. from University College London, investigated Internet use and rural-urban mental health inequalities in China. Their research provides valuable insights into the role of internet usage in shaping mental health disparities between urban and rural populations.

Subsequently, addressing potential negative experiences that may arise from internet usage, this Research Topic examined the relationship between cyberbullying, internet addiction, and mental health. A systematic review by Jeyagobi et al. from Malaysia examined the factors influencing negative cyber-bystander behavior and its association with mental health issues. Their research provides insights into bystander dynamics and the potential effects on mental wellbeing, shedding light on cyber-aggression's broader impact. Zhang et al. from Anhui, China explored the bidirectional association between smartphone addiction and depression among college students. Their findings underscore the need for comprehensive interventions addressing both smartphone addiction and depression to improve mental health outcomes.

As one of the most crucial products of the digital era, the influence of social media on mental health cannot be overlooked. Bonsaksen et al. from Norway conducted a study to illuminate the intricate link between adolescents' engagement with social media and their mental wellbeing. The findings underscore the critical role of understanding and mitigating negative digital experiences for safeguarding adolescent mental health in the age of pervasive social media engagement. Kim et al.'s study delved into COVID-19-related anxiety and the role of social media among Canadian youth. Their research highlights the interplay between social media usage and mental health during the pandemic, raising awareness of potential risks and benefits for youth mental wellbeing. Moreover, in a thought-provoking perspective article, Leightley et al. proposed a framework to maximize the positive and minimize the negative impact of social media data in studying youth mental health. With multidisciplinary perspectives from authors affiliated with various institutions, their work addresses the importance of secure data access while promoting mental health research in the digital age.

Following that, this Research Topic delved into the application and effectiveness of emerging technologies in the field of mental health treatment. Roncero et al. from Spain presented an original research study exploring healthcare professionals' perception of and satisfaction with mental health tele-medicine during the COVID-19 outbreak. Their work showcases the potential of telepsychiatry to improve mental health care accessibility and highlights its relevance in addressing mental health challenges during crises. Funnell et al. from Cambridge, United Kingdom investigated user feedback of a novel digital mental health assessment. Their study offers valuable insights into user experiences and preferences, contributing to the development of effective and user-friendly digital mental health interventions. In addition, research by Ho et al. from Hong Kong Polytechnic University and co-authors examined heart rate variability as a potential biomarker for hope, contributing to a deeper understanding of the digitization and visualization of mental wellbeing and its possible objective assessment using bio-indices.

By exploring these multifaceted dimensions of the topic, this Research Topic aims to deepen our understanding of how emerging technologies and products can impact mental health and provides valuable insights for researchers, clinicians, and policymakers in developing evidence-based interventions and promoting mental wellbeing in the digital age. The articles presented herein underscore the need for continued research, thoughtful policymaking, and responsible usage to strike a delicate balance between embracing technological progress and safeguarding our mental wellbeing.

## Author contributions

CG: Conceptualization, Funding acquisition, Writing—original draft, Writing—review and editing. SG: Writing—original draft, Writing—review and editing. PS: Writing—review and editing. AF: Writing—review and editing.
